# Attitudes of Bedouin and Jewish Physicians Towards the Medical Care for Persons with Intellectual Disability in the Bedouin Negev Community. A Pilot Study

**DOI:** 10.1100/tsw.2004.125

**Published:** 2004-08-13

**Authors:** Mohammed Morad, Tagrid Morad, Isack Kandel, Joav Merrick

**Affiliations:** ^1^ Clalit Health Services and Division of Community Health, Department of Family Medicine, Ben Gurion University, Beer-Sheva, Israel; ^2^ National Institute of Child Health and Human Development, Office of the Medical Director, Division for Mental Retardation, Ministry of Social Affairs, Jerusalem and Zusman Child Development Center, Division of Pediatrics and Community Health, Ben Gurion University, Beer-Sheva, Israel; ^3^ Faculty of Social Sciences, Department of Behavioral Sciences, Academic College of Judea and Samaria, Ariel, Israel

**Keywords:** intellectual disability, developmental disability, mental retardation, Bedouin, human development, public health, Israel

## Abstract

Change in the attitudes of staff or the public towards people with intellectual disability (ID) can impact their life and health, but that change has not been studied among physicians who belong to an ethnic minority undergoing dramatic social and economic transition. The goal of this study was to explore the change of attitudes of Negev Bedouin physicians serving their community and their satisfaction with policy, care, and knowledge in the field of ID. Seventeen community physicians (7 Bedouins and 10 Jewish) were interviewed using a simple questionnaire that consisted of items measuring attitude and satisfaction. The vast majority of the Bedouin and Jewish physicians had positive attitudes toward inclusion of those in the community with ID and were ready to provide the care needed in the community with special assistance. There was a need for further education in ID and more resources. There was a belief that there is discrimination between the Bedouin and Jewish community in the provision of care to people with ID. General dissatisfaction was expressed about the policy, resources, care provision, and expertise offered to Bedouins with ID. More efforts must be directed to empower the physicians with knowledge, expertise, and resources to handle the care of Bedouins with ID in a culturally appropriate way.

## INTRODUCTION

Bedouin physicians have started sharing the health care of the Negev Bedouin community. Many of these physicians graduated abroad at western or eastern faculties of health in order to continue their specialization in Israeli medical centers. According to local service provider information[1], most of them (21 out of 25) were employed as family physicians, general practitioners, or pediatricians and were mainly involved in the care of the Bedouin community (2 in the unplanned settlements and 19 in the planned Bedouin towns) at the Clalit Health Service (HMO) clinics, at their own private clinics, and clinics for maternal and child care. Bedouin physicians have been exposed to the western value system and Internet at work, using e-mail and on-line databases and journals.

Even though the Bedouin community and the Arab community in general in Israel[2] are under transition towards a western culture, the community is still characterized by polygamy, consanguineous marriages, extended family loyalty, and persisting tribal life elements that include low socioeconomic status of women, children, disadvantaged mentally ill, and the disabled[3]. This community is mainly characterized by poverty, unemployment, school drop-out, lack of social resources, high fertility rate, and increased infant mortality[4].

In the 1990s, more than 300 Bedouins with intellectual disability (ID) were registered at the social welfare authorities in the Negev, with most of them in the community due to a traditional family system and fear of stigma, but also because of the lack of residential care facilities in the vicinity[5]. The number of persons with ID would be excepted to increase due to the high fertility rate, the higher rate of consanguinity, and genetic diseases among the Negev Bedouins.

The attitude of general practitioners towards caring for persons with ID has been investigated in Wales[6] with a postal questionnaire and response from 126 GPs, who generally agreed that GPs should be responsible for the medical care of people with ID, but they tended to oppose the provision of regular structured health promotion, such as annual health check-up, assessing hearing or eyesight. Another study from Australia[7] looked at the attitude among GPs to address nine specified health care issues (regular hearing and vision assessment; regular dental care; review of medication; thyroid testing in Down syndrome; health screens like pap smear, mammography, or blood pressure; nutritional advice; lifestyle advice; psychiatric disorders; and a comprehensive annual health review). This questionnaire was mailed to a random sample of all Australian GPs and 529 responded. They showed a more positive attitude concerning the role and responsibilities of the GP in providing health care to people with ID than previous studies. It seemed that the questions generated an awareness and intention to increase these issues concerning their patients.

The attitudes of Arab social work staff toward integration of people with ID in the community has been investigated[8], but attitudes among physicians or Negev Bedouin physicians have so far not been studied.

In this pilot study, we will focus on the attitudes of Bedouins physicians compared to Jewish counterparts, look at their satisfaction with the services provided to the Bedouins compared to those provided to the Jewish community in the Negev, and explore the quality of attitudes and the satisfaction of Bedouin physicians toward the care provided by them and others to Bedouin persons with ID compared to that provided to the Jewish patients with ID in the Negev.

## METHODS

A simple questionnaire was developed about attitudes and satisfaction of the care provided to persons with ID, demographic data, and information on place of study, education, specialization, and expertise in the field of ID. The questions were based on core concepts of normalization and recommendations of scientific associations suggesting integration of people with ID in the community, while providing them with high quality primary care supported by specialist care.

We found it important to include the issues of empowerment and preservation of the rights of those with ID in the attitude section of the questionnaire. Issues of satisfaction of the physician cover many concerns regarding insufficient expertise, lack of resources at the primary care setting, and the inappropriateness of some care aspects such as consultation and diagnostic tools.

We used a snowballing technique to survey our sample, but when e-mail was not possible, face-to-face interviews were used. A pretest was conducted to test the questionnaire with an interview of eight physicians, four Bedouins and four Jewish. Some changes were made in the process. We used a questionnaire in Hebrew as suggested by the pilot group.

Completed forms were returned in less than 1 week, without a need for reminders. The questionnaires were returned by mail, fax, or e-mail and the data were analyzed by SPSS version 11.

## RESULTS

The questionnaire including 22 items on attitudes and satisfaction of the care of Bedouins with ID was sent by e-mail to 24 Bedouin and Jewish physicians providing care at the Clalit Health Service (HMO) clinics and to 4 health policy makers and HMO administrators. In cases of delay or nonresponse, a face-to-face meeting with the physician was a second option and there was a very high response rate (17/24).

**TABLE 1**
**Demographics of the Sample Studied**

| | No. | Males | Females | Mean Age | Study in Israel | Pediatricians | Family Physicians | GPs | Other |
|---|---|---|---|---|---|---|---|---|---|
| Arabs | 7(41%) | 6 | 1 | 40.42 | 1 | 3 | 3 | 1 | 0 |
| Jews | 10(59%) | 8 | 2 | 48.7 | 3 | 3 | 2 | 1 | 4 |

All Bedouin and Jewish physicians interviewed agreed that most people with ID should be included in the community, while only 82% expressed their agreement to give medical care at the community clinics. All physicians agreed that people with ID deserve universal health care equal to the general population with the addition that they have to receive special care for their unique needs and health problems.

The majority of the participating physicians in the study (88%) wanted care providers and the Ministry of Health to provide more time and resources to persons with ID. They also felt that new technologies and drugs developed specifically to meet the needs of people with ID should be provided to this population. The majority (94%) also had a positive attitude toward accountability of the care and service provided and felt that people with ID could express their needs, expectations, attitudes, and satisfaction with the care provided to them. Informed consent is an essential and eminent component in the care process for persons with ID according to the attitude of majority of the study participants (84%).

Another large percentage (94%) of the physicians expressed the need for special qualification in ID sharing the care for this population at community settings. All participants agreed that each physician should be qualified to optimally communicate, verify the complaints, and to refer any person with ID to other special care providers. All physicians felt that a positive attitude towards persons with ID should be elementary in order to improve the care, satisfaction, and strengthen the care provider-client contact.

Of the participants, 76% considered the care for persons with ID in the Bedouin community to be worse than that offered to Jewish persons with ID and 88% of the interviewed physicians found that the law assuring equal rights for both populations was not implemented. The majority of participants (76%) said that the gap between the care for Bedouins and Jews originated from budgetary, national, and political reasons, and 82% of the physicians were not satisfied with the care offered to Bedouins with ID compared to Jews, including different aspects of care such as diagnostic services (76%), while most of them expressed displeasure with the health policy for the Bedouins with ID compared to Jews (76%). As they were convinced that the budgets were not enough to meet the needs of people with ID, physicians expressed their discontent with this budget deficit (70%). They openly expressed their disappointment with the insufficient knowledge and expertise of physicians in the field of ID (76%) and the deficit in the consultation offered to parents with children with ID (70%). Only half of the physicians were happy with their own expertise in order to handle the care for persons with ID. Nationality was correlated with satisfaction about knowledge and expertise of the physician, where Jewish physicians were more satisfied with their own knowledge and expertise while caring for people with ID in both sectors, but it could be related to the older age of Jewish doctors. Older physicians were also more satisfied than the younger physicians in regards to diagnostic services, budget, and resources available for persons with ID. Most of the young doctors were Bedouins.

Where the physician had studied was correlated with a positive attitude toward inclusion of persons with ID in the community, as studying abroad tended to shift these attitudes to be positive irrespective of being Bedouin, female, older, or the physician’s specialty. Studying abroad correlated negatively with the level of satisfaction about their own knowledge and expertise in the field of ID. Pediatricians expressed more satisfaction with their own knowledge and expertise.

In their responses, physicians demanded that more professional health attention should be given to people with ID. They also asked for more help and guidance by qualified physicians in ID medicine in order to share the burden of primary care for this population. Physicians in primary care agreed to provide health care in community clinics, but they were critical regarding accountability for health care and less satisfied with their knowledge and expertise. Primary care physicians were less satisfied with their own performance as service providers for persons with ID and expressed the importance of counseling families and parents.

Responders who believed in discrepancies between the care for Bedouins with ID and Jews with ID were less satisfied with the policy implementation among Bedouins and Jews. The dissatisfaction with the health care was general and included different aspects of the care.

## DISCUSSION

Health policy makers[9] have recently held discussions on developing community welfare systems to serve the Negev Bedouins and mentioned the possibility that this process could be easier, since Bedouins have not been exposed to the institutional model of care to a large extent. Providing an effective health service to Bedouins with ID should take into consideration the difficult reality of Bedouin life and family, the lack of resources, the status of the child in the tribal system, and the patriarchal type of the Bedouin family.

Physicians are not the only professionals involved with the Bedouin population. Nurses, administrators, allied health professionals, and social workers are also eminent parts of such a transition from mostly informal care to formal modern western type of care[10]. Recent work from the United Arab Emirates[11] provided evidence about the possible involvement of culture and socioeconomic factors in the development of psychiatric problems among children with ID and another study from Israel showed differences in the patterns of utilization of rehabilitation services, most probably affected by social, economic, and cultural factors[10]. The interwoven factors coming from informal support systems, transition features of society, and marital and economic stress were clearly demonstrated in a recent study of stress, social support, and the well being of Arab mothers of children with ID[12], but research on the contribution of change of attitudes of health professionals and the impact on the person with ID in the “embedded Arab family in the traditional society with extended family support systems under transition” is still pending.

The attitude of the Negev Bedouin physician had not been explored in general before and not at all toward the issue of care of the intellectually disabled[8]. Our small study confirmed that Bedouin physicians, as was seen among Arab social workers in Northern Israel[8], have changed their traditional attitudes toward people with ID and these positive attitudes will be very important to provide the needed culturally appropriate care to persons with ID in the Bedouin sector in the Negev. This is important, because the positive attitude toward persons with ID may improve their care, satisfaction, and quality of life. Early exposure and exposure during educational activity or work can positively affect the attitudes of persons toward people with ID[13,14], but the persistence of these positive attitudes remains controversial[15].

Our study found the need for further education of primary health care workers in the field of ID, which they share with many professionals around the world who lack the needed expertise and assistance from specialists, but who otherwise are ready to provide care for these people in the community[16,17]. Our study, in a very clear message, provided the evidence that the physicians' attitudes and satisfaction regarding the care for the intellectually disabled are interrelated regardless of ethnicity, exposure to different culture, specialty, or education.

## CONCLUSION

Bedouin doctors are ready to provide care for the intellectually disabled in the community, but think that they need more budgets, expertise, and help from other health specialists to make the care effective and comprehensive. They believe that even though the law assures equal rights for the disabled in Israel, there are many political, national, and economic issues that should be resolved in order to make the service for the Bedouins comparable to that provided to the Jewish community.

They share the positive attitudes toward people with ID with their Jewish colleagues and, unfortunately, also the low satisfaction with the care provided. They are more in need than their Jewish counterparts for education in the field of ID because of the minimal exposure to this issue during their studies and their low satisfaction with expertise in ID.

The was agreement between Jewish and Bedouin physicians that the budget and barriers are still blocking the provision of appropriate care compared those in the Jewish population with ID, which signalizes a need to involve them in the process of planning, providing, and evaluating the services for the Bedouins with ID.

## BIOSKETCHES

**Mohammed Morad, MD**, is Specialist in Family Medicine, Lecturer in Family Medicine at the National Institute of Child Health and Human Development, Division of Community Health, Ben Gurion University of the Negev and the Medical Director of a large area clinic in the city of Beer-Sheva. He has publications on Bedouin health, health aspects, spiritual health, and aging in persons with intellectual disability, and is a presenter on topics such as health policy and services for the disadvantaged at national and international conferences. E-mail: morad62@barak-online.net

**Tagrid Morad,** High School Student and Research Assistant at the National Institute of Child Health and Human Development. E-mail: morad62@013.net.il

**Isack Kandel**, **MA, PhD**, is Senior Lecturer at the Faculty of Social Sciences, Department of Behavioral Sciences, the Academic College of Judea and Samaria, Ariel. During the period 1985–1993, he served as the Director of the Division for Mental Retardation, Ministry of Social Affairs, Jerusalem, Israel. E-mail: Kandeli@aquanet.co.il

**Joav Merrick, MD, DMSc,** is Professor of Child Health and Human Development affiliated with the Zusman Child Development Center, Division of Pediatrics and Community Health at the Ben Gurion University, Beer-Sheva, Israel; the Medical Director of the Division for Mental Retardation, Ministry of Social Affairs, Jerusalem; and the Founder and Director of the National Institute of Child Health and Human Development. He has numerous publications in the field of child and human development, rehabilitation, intellectual disability, disability, health, welfare, abuse, advocacy, quality of life, and prevention. Dr. Merrick received the Peter Sabroe Child Award for outstanding work on behalf of Danish Children in 1985 and the International LEGO-Prize ("The Children's Nobel Prize") for an extraordinary contribution towards improvement in child welfare and well being in 1987. E-mail: jmerrick@internet-zahav.net. Website: www.nichd-israel.com
